# *Azolla filiculoides* extract improved salt tolerance in wheat (*Triticum aestivum* L.) is associated with prompting osmostasis, antioxidant potential and stress-interrelated genes

**DOI:** 10.1038/s41598-024-61155-7

**Published:** 2024-05-15

**Authors:** Asma A. Al-Huqail, Nagwa M. A. Aref, Faheema Khan, Sherien E. Sobhy, Elsayed E. Hafez, Asmaa M. Khalifa, Khalil M. Saad-Allah

**Affiliations:** 1 Chair of Climate Change, Environmental Development, and Vegetation Cover, Department of Botany and Microbiology, College of Science, King Saud University, 11451 Riyadh, Saudi Arabia; 2 Department of Microbiology, Faculty of Agriculture, Ain Shams University, Hadayek Shubra 11241, Cairo, Egypt; 3 Plant Protection and Bimolecular Diagnosis Department, Arid Lands Cultivation Research Institute, City of Scientific Research and Technological Applications, New Borg El‑Arab, 21934 Egypt; 4 Botany and Microbiology Department, Faculty of Science, Al Azhar University (Girls Branch), Cairo, 71524 Egypt; 5 Botany Department, Faculty of Science, Tanta University, Tanta, 31527 Egypt

**Keywords:** *Azolla* extract, Salt stress, Wheat growth, Oxidative balance, Molecular response, Physiology, Plant sciences

## Abstract

The growth and productivity of crop plants are negatively affected by salinity-induced ionic and oxidative stresses. This study aimed to provide insight into the interaction of NaCl-induced salinity with *Azolla* aqueous extract (AAE) regarding growth, antioxidant balance, and stress-responsive genes expression in wheat seedlings. In a pot experiment, wheat kernels were primed for 21 h with either deionized water or 0.1% AAE. Water-primed seedlings received either tap water, 250 mM NaCl, AAE spray, or AAE spray + NaCl. The AAE-primed seedlings received either tap water or 250 mM NaCl. Salinity lowered growth rate, chlorophyll level, and protein and amino acids pool. However, carotenoids, stress indicators (EL, MDA, and H_2_O_2_), osmomodulators (sugars, and proline), antioxidant enzymes (CAT, POD, APX, and PPO), and the expression of some stress-responsive genes (POD, PPO and PAL, PCS, and TLP) were significantly increased. However, administering AAE contributed to increased growth, balanced leaf pigments and assimilation efficacy, diminished stress indicators, rebalanced osmomodulators and antioxidant enzymes, and down-regulation of stress-induced genes in NaCl-stressed plants, with priming surpassing spray in most cases. In conclusion, AAE can be used as a green approach for sustaining regular growth and metabolism and remodelling the physio-chemical status of wheat seedlings thriving in salt-affected soils.

## Introduction

Climate change poses a severe threat to life on Earth, particularly through alterations in soil characteristics, groundwater levels, microbial communities, and crop physiology. These changes have a detrimental impact on plant yields and subsequently jeopardize food security. Soil salinity, a complex stress factor resulting from insufficient precipitation, high evaporation rates, poor drainage, misuse of chemical fertilizers, and saline-polluted water, poses a significant environmental challenge^1^. Insufficient precipitation rates, elevated levels of evaporation, poor drainage, improper use of chemical-based agricultural fertilizers, and watering with saline-polluted water are the main factors contributing to excessive salt concentrations in agricultural soils^2^. The resulting excessive salt concentrations in agricultural soils interfere with plant development and productivity, leading to growth deterioration and a cascade of physiological and biochemical implications^3^. One such consequence is the generation of ionic and osmotic disturbances, which impede plant growth and biomass yield. Moreover, salt-induced injuries manifest before visible damage symptoms appears on leaves, as plants experience osmotic stress, ionic stress, nutrient insufficiency, and other physiological disruptions. High salt concentrations trigger a range of adverse effects, including decreased growth and biomass yield, chlorophyll degradation, protoplasm dehydration, impaired transpiration and respiration rates, inhibited cell division and expansion, enzymatic activity inhibition, membrane malfunction, altered gene expression, and disrupted ionic homeostasis^4–6^. Additionally, the presence of activated oxygen species leads to oxidative stress, damaging nucleic acids, proteins, and membrane lipids, further compromising cell and organelle functionality and resulting in decreased growth and yield^7,8^.

Various approaches have been developed to alleviate salt stress in plants, such as seed priming, foliar spray, and genetic manipulation. While some methods rely on chemical agents, recent studies have explored naturally occurring, cost-effective, environmentally friendly, and profitable alternatives. Natural extracts derived from plants, algae, and microorganisms have demonstrated the ability to enhance plant growth and yield under stress conditions, while mitigating the drawbacks associated with synthetic substances. Noteworthy examples include the use of *Arthrocnemum macrostachyum* to reduce salt effects in soybean^9^, sorghum water extract for camelina^10^, moringa leaf extract for common bean^11^, and *Ulva lactuca* extract for tomato^12^. These extracts exhibit beneficial properties linked to their ability to reduce stress-related oxidative damage, regulate antioxidant activities, accumulate non-enzymatic antioxidants, maintain nutritional homeostasis, prevent ion leakage, promote membrane consistency and fluidity, and stimulate the accumulation of osmoprotectants like proline, glycinebetaine, sugars, and amino acids^13–15^.

Among aquatic macrophytes, *Azolla* species have been identified as economically valuable due to their rapid growth rate and the symbiotic relationship between *Azolla* leaves and the cyanobacterium *Anabaena azolae*, which enables nitrogen fixation^16^. *Azolla* serves as a natural supplier of nitrogen for agricultural and livestock production, capable of fixing 30–60 kg/h^−1^ of nitrogen. Furthermore, Azolla enhances the nutritional value of rice in paddy fields and reduces the need for urea fertilizer^17^. It also supplies vitamins, growth stimulants, essential amino acids, growth-promoting intermediaries, and mineral ions (e.g., Ca, Mg, K, P, Fe, and Cu)^18^. Three Azolla species, namely *A. pinnata, A. filiculoides,* and *A. Africana*, show potential as nitrogen sources for crops^16^. Although *A. filiculoides* can obstruct irrigation canals, it has proven to be an effective biofertilizer for various crop species under different conditions, including maize under water stress and nitrogen deficiency^18^, wheat in drought-affected soil^19^, and chard in hydroponics^20^.

Wheat (*Triticum aestivum* L.), a Poacea staple crop grown worldwide, is characterized by its adaptability, substantial yield, nutritional value, and industrial applications. Wheat is distinguished by its high concentration of dietary fibers, essential amino acids, minerals, and vitamins, as well as other phytochemicals^21^. However, wheat, like many other plant species, is susceptible to salt-related stress due to a lack of a well-developed defensive mechanism^22^. As a result, a practical approach is needed to appropriately counter the adverse impacts of salt stress on plant metabolic processes. External administration of growth stimulants is one method used to reduce the damaging effects of salt stress on plants^4^. We, therefore, seek to assess the effectiveness of *A. filiculoides* aqueous extract (AAE) as both a priming treatment and a foliar administration in mitigating the adverse effects of salt stress on the growth, metabolic performance, and molecular responsiveness of wheat plants exposed to high salt concentrations (250 mM NaCl). The study also aimed to determine whether the AAE extract contains specific phytochemicals that can mitigate salt-induced toxicity in wheat plants and elucidate the underlying mechanisms through which these phytochemicals alleviate salt toxicity.

## Results

### Growth attributes

**Figure 1:**
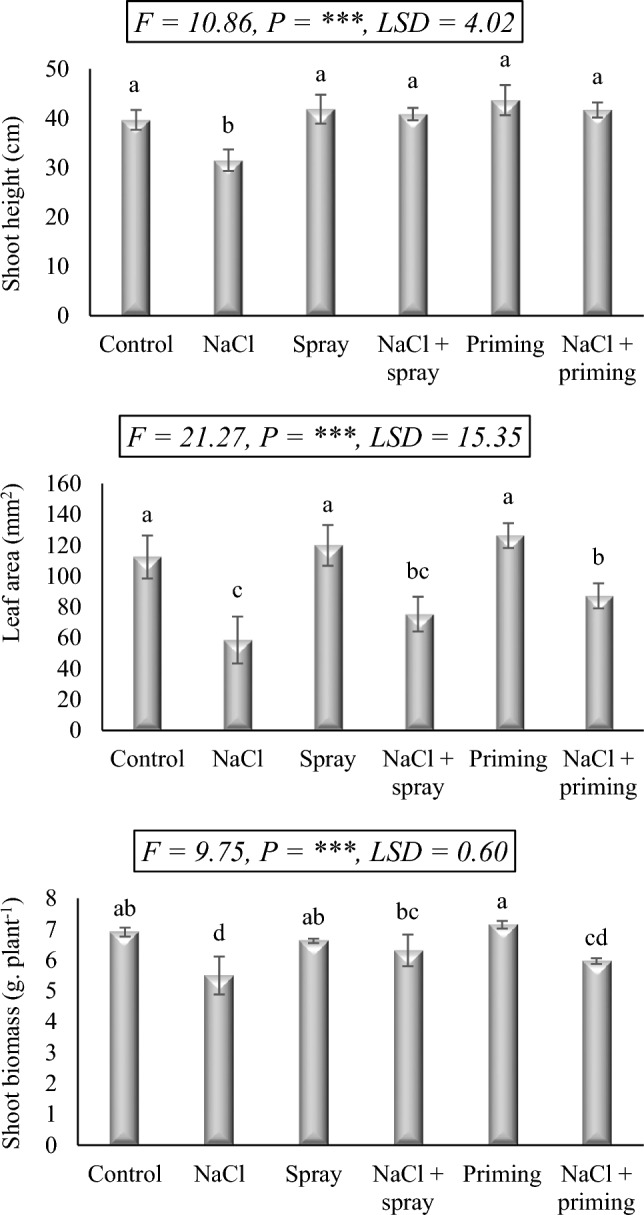
Effect of priming and foliar application of *Azolla* aqueous extract (AAE) on the growth attributes of wheat seedlings subjected to salt stress. Different letters donate significant differences at 0.05 level using the Post Hoc Duncan test. *F* Fisher's test for determining significant variance between treatment means, *P* probability, *LSD* least significant difference at 5% level.

The data displayed in Fig. 1 reflects the response of wheat (*Triticum aestivum* L. cv Sakha 96) to salt stress (250 mM NaCl), *Azolla* aqueous extract (AAE), foliar and priming treatments, either alone or in combination with the salt treatment. The findings demonstrated that salt treatment has a detrimental effect on the assessed growth characteristics of wheat seedlings. In comparison to the equivalent control values, salt treatment significantly decreased the shoot height, leaf area, and shoot biomass by 20.6, 47.9, and 20.3%, respectively.

AAE treatments, nonetheless, exhibited a favorable impact on the examined parameters, surpassing control values in most assessments, particularly when utilized as a priming treatment. In comparison with salt treatment, AAE significantly enhanced the growth performance of salt-stressed wheat seedlings compared to individual salt treatment. These combinations proved effective in augmenting shoot biomass and leaf area in the stressed plants, although still lower than those of the control. Notably, AAE treatments resulted in shoot height surpassing that of the non-stressed control, highlighting the notable improvement in shoot height. Consequently, based on our findings, the administration of AAE may be regarded as an efficacious strategy for promoting the survival of wheat plants in salt-affected regions.

### Leaf pigmentation and photosynthetic performance

**Figure 2:**
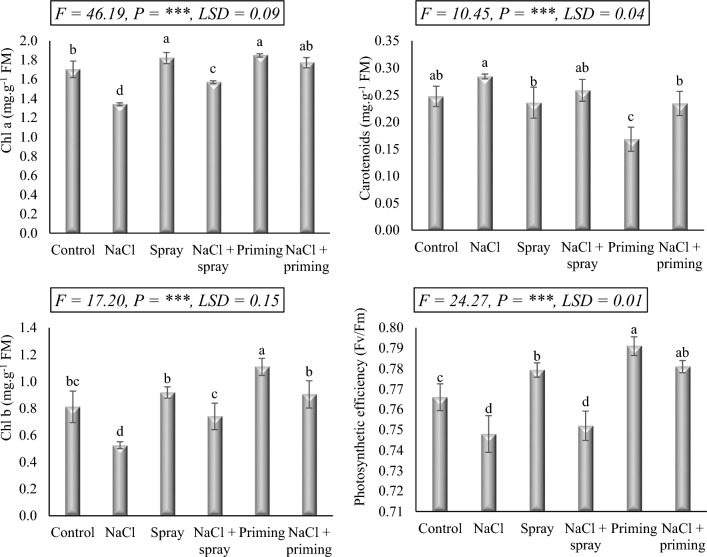
Effect of priming and foliar application of *Azolla* aqueous extract (AAE) on leaf pigments and photosynthetic activity of wheat seedlings subjected to salt stress. Different letters donate significant differences at 0.05 level using the Post Hoc Duncan test. *F* Fisher's test for determining significant variance between treatment means, *P* probability, *LSD* least significant difference at 5% level.

The photosynthetic pigments content of wheat leaves as well as their photosynthetic performance (Fv/Fm) were significantly affected by the treatments used in this investigation (Fig. 2). Comparatively, the exposure of wheat seedlings to 250 mM NaCl led to a significant reduction in Chl a and Chl b contents (21.2, and 35.3%, respectively), as compared the to control values. Moreover, among the treatments applied, the Fv/Fm value of salt-stressed wheat seedlings exhibited the lowest value, experiencing a 2.4% decline from the control. Conversely, NaCl treatment resulted in the highest carotenoid content in this investigation, displaying a 14.5% increase compared to the control. Interestingly, both priming and foliar administration of AAE demonstrated a positive impact on Chl a and Chl b fractions, particularly Chl b in the case of priming treatment, surpassing control levels. Similarly, Fv/Fm of wheat improved due to AAE administration, particularly with the priming treatment.

When AAE and NaCl were co-administered, as opposed to NaCl treatment alone, the adverse effects on Chl a and Chl b levels, as well as Fv/Fm, were ameliorated. In many cases, the combined interactions exceeded the control threshold, particularly when AAE was used as a priming treatment alongside salt treatment. Regarding carotenoids, the excessive levels induced by salt application were rectified by AAE administration, particularly when AAE was employed as a priming treatment prior to salt stress exposure in wheat seedlings.

### Oxidative injury markers

**Figure 3:**
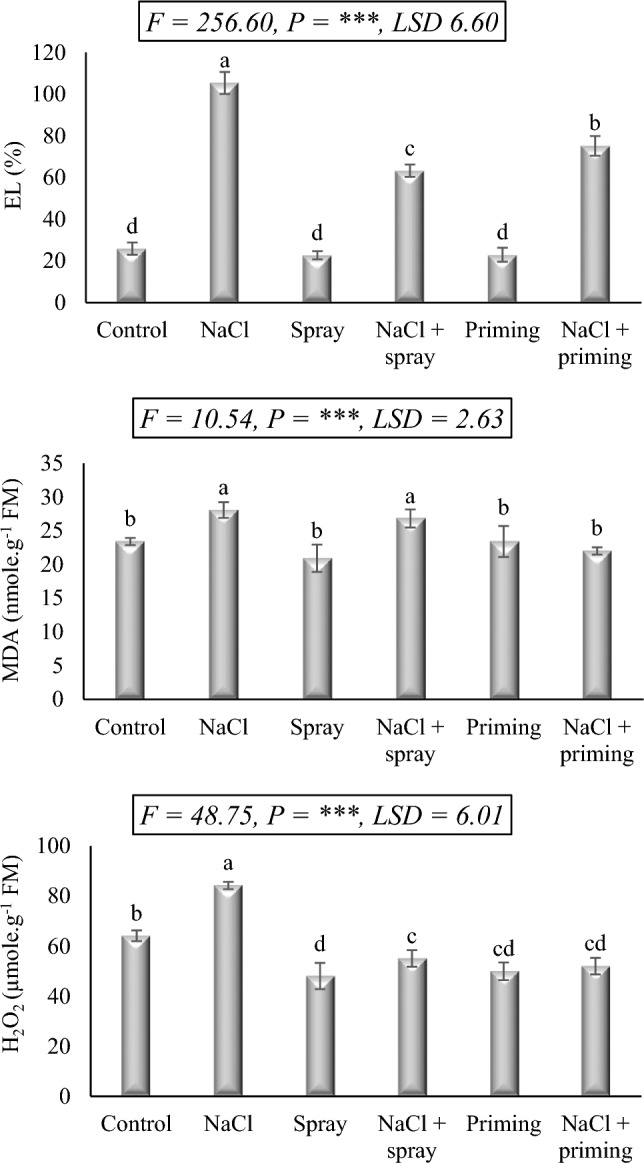
Effect of priming and foliar application of *Azolla* aqueous extract (AAE) extract on stress biomarkers of wheat seedlings subjected to salt stress. Different letters donate significant differences at 0.05 level using the Post Hoc Duncan test. *F* Fisher's test for determining significant variance between treatment means, *P* probability, *LSD* least significant difference at 5% level.

Salt exposure has a secondary impact on biological membranes, whereby an excessive presence of active oxygen species leads to the oxidation of proteins and phospholipids, resulting in the loss of selective permeability and uncontrolled ion leakage. Figure 3 illustrates the detrimental effects of NaCl exposure on wheat leaves, characterized by elevated electrolyte leakage (EL), lipid peroxidation measured by malondialdehyde (MDA) content, and excessive hydrogen peroxide (H_2_O_2_) production. The increases in EL, MDA, and H_2_O_2_ levels in wheat leaves, induced by NaCl application, reached 306.9, 19.9, and 31.3%, respectively, in comparison to the control values. Notably, the utilization of AAE, whether as a priming treatment or foliar application, significantly mitigated oxidative stress markers in wheat seedlings, evident through reduced EL, MDA, and H_2_O_2_ levels, frequently below those of the control treatment.

The results highlight the apparent restorative response of AAE in alleviating stress markers in wheat leaves under salt stress. Comparative analysis revealed that the application of AAE, either as priming treatment or foliar spray, decreased EL levels in salt-stressed wheat seedlings. Significantly, when AAE and NaCl were administered together, the H_2_O_2_ content in the stressed seedlings decreased to levels lower than those observed in the control. However, the combination of AAE foliar spray and salt treatment unexpectedly showed a non-significant decrease in MDA content compared to the salt treatment, while the combination of AAE priming and salt treatment effectively reduced the excessively accumulated MDA.

### Osmomodulatory metabolites

**Figure 4:**
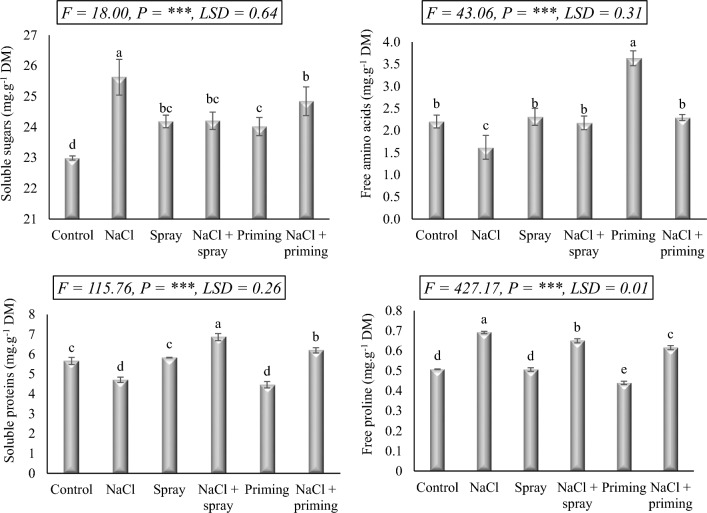
Effect of priming and foliar application of *Azolla* aqueous extract (AAE) on osmomodulatory molecules level of wheat seedlings subjected to salt stress. Different letters donate significant differences at 0.05 level using the Post Hoc Duncan test. *F* Fisher's test for determining significant variance between treatment means, *P* probability, *LSD* least significant difference at 5% level.

The ionic stress developed by salinity exposure could give rise to analogous modifications in the osmotic equilibrium of the stressed plants. The concentration of soluble sugars (SS), soluble proteins (SP), free amino acids (FAA), and free proline (FP) showed a clear fluctuation as affected by the experimental treatments (Fig. 4). In this study, exposure to NaCl stress led to a significant increase in SS and FP levels, elevating their concentrations by 11.4 and 38.0%, respectively, compared to the control. Conversely, NaCl treatment resulted in a decrease of 17.8% in SP and 26.4% in FAA. The applied treatments of AAE displayed more or less enhancement in the concentrations of SS and FAA, with the priming treatment showing a more pronounced increase in FAA levels. Regarding SP and FP levels, foliar application of AAE on wheat did not show any discernible effect, while the priming treatment led to a relative decrease in their concentrations compared to the control. The levels of osmomodulatory metabolites evaluated in salt-stressed plants were restored upon AAE administration. According to the data provided, pre-treatment with AAE (priming) or post-treatment with AAE (foliar spray) restored the levels of the aforementioned metabolites to values that were relatively close to those of the control, particularly FAA.

### Enzymes activity

**Figure 5:**
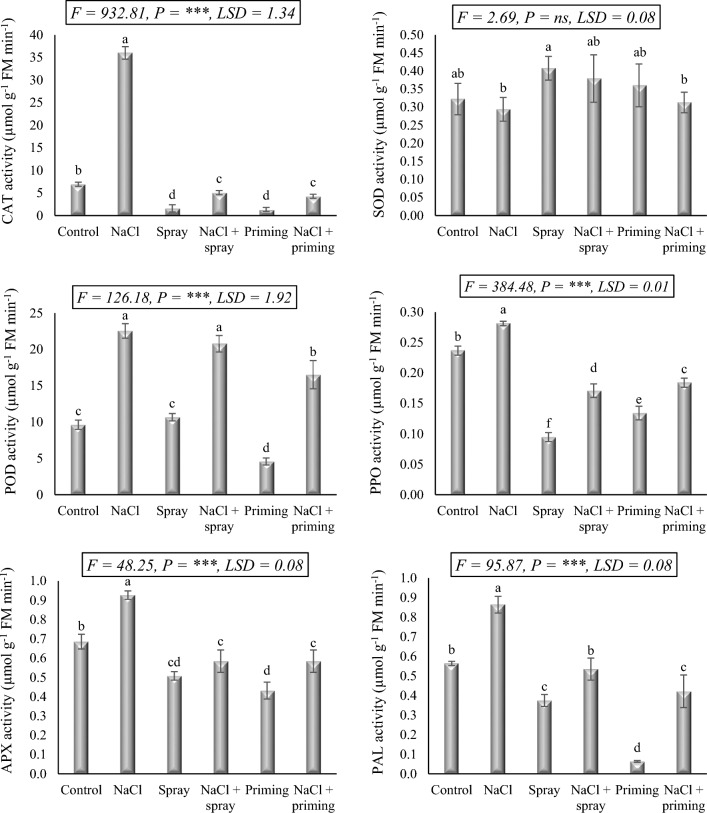
Effect of priming and foliar application of *Azolla* aqueous extract (AAE) on antioxidant enzymes activity of wheat seedlings subjected to salt stress. Different letters donate significant differences at 0.05 level using the Post Hoc Duncan test. *F* Fisher's test for determining significant variance between treatment means, *P* probability, *LSD* least significant difference at 5% level.

As seen from Fig. 5, salt and AAE treatments significantly altered the activities of catalase (CAT), guaiacol peroxidase (POD), ascorbate peroxidase (APX), superoxide dismutase (SOD), polyphenol oxidase (PPO), and phenylalanine ammonia-lyase (PAL). Except for SOD, the administered salt treatment elicited an increase in the activities of the assayed enzymes. CAT, POD, APX, PPO, and PAL activities were significantly enhanced by 419.5, 134.2, 35.3, 18.6, and 53.6%, respectively, in response to salt treatment. Conversely, SOD activity showed a non-significant decrease of 9.0% in response to the applied NaCl dosage. The treatments of AAE, particularly the priming treatment, effectively attenuated the activities of these enzymes (CAT, POD, APX, PPO, and PAL) to levels comparable to control plants. Furthermore, AAE administration induced a non-significant stimulation in SOD activity.

When salt and AAE treatments were administered simultaneously, with priming treatment demonstrating greater efficacy than foliar application, the interactive combination significantly reduced the salt-induced increase in the activities of CAT, APX, PPO, and PAL. These reductions generally reached levels below that of the control in most combinations. POD activity decreased below that of stressed plants due to the combined effects of salt treatment and AAE, but it remained higher than the control activity. However, the activity of SOD exhibited minor fluctuations, decreasing or increasing in response to the combined interaction of AAE and NaCl.

### qRT-PCR pattern of the investigated genes

**Figure 6:**
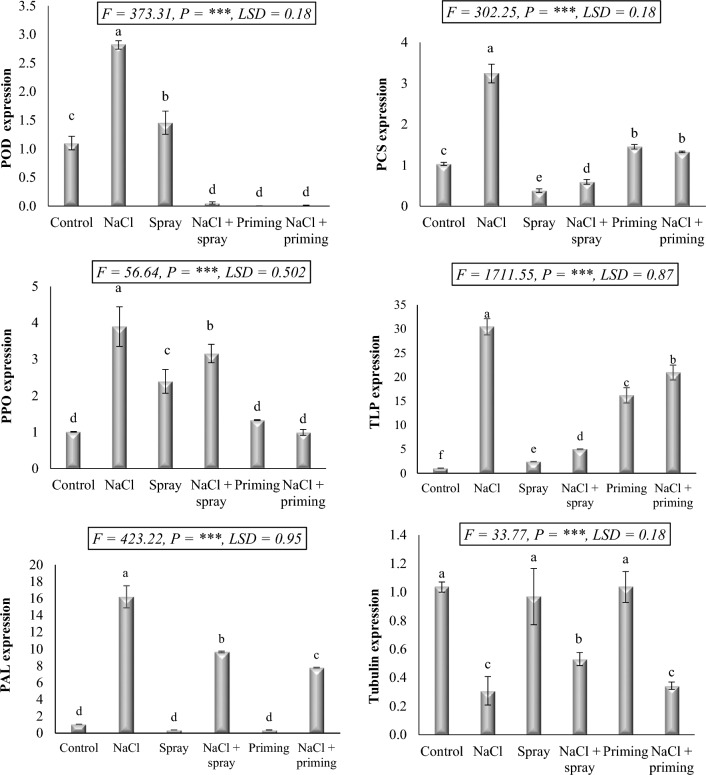
Effect of priming and foliar application of *Azolla* aqueous extract (AAE) on the stress-responsive genes expression level of wheat seedlings subjected to salt stress. Different letters donate significant differences at 0.05 level using the Post Hoc Duncan test. *F* Fisher's test for determining significant variance between treatment means, *P* probability, *LSD* least significant difference at 5% level.

When wheat is grown in a saline environment, the expression of certain genes that permit it to withstand such stressful conditions plays a significant role in its development, metabolic response, and yield. In this study, we investigated the impact of AAE application, either through priming or foliar treatment, on the expression of six enzymes in wheat seedlings, namely peroxidase (POD), polyphenol oxidase (PPO), phenylalanine ammonia-lyase (PAL), phytochelatin synthase (PCS), thaumatin-like protein (TLP), and tubulin (Fig. 6). Under salt stress conditions, our results revealed that 250 mM NaCl significantly upregulated the expression of POD, PPO, PAL, PCS, and TLP (2.56, 3.86, 15.47, 3.13, and 29.48-fold, respectively). In contrast, the expression pattern of tubulin was downregulated by 3.36-fold due to salt treatment. Application of AAE as a foliar spray in wheat led to a significant increase in the expression of POD (1.32-fold), PPO (2.37-fold), and TLP (2.34-fold), while decreasing the expression of PAL, PCS, and tubulin (2.79, 2.72, and 1.06-fold, respectively). Priming with AAE resulted in decreased expression of POD and PAL (9.08 and 2.08-fold, respectively), increased expression of PPO, PCS, and TLP (1.32, 1.40, and 15.68-fold, respectively), with no noticeable change in tubulin expression. The interaction between salt and AAE treatments resulted in diverse alterations in the expression of the investigated genes. Notably, the combination of priming or foliar application AAE with NaCl treatment led to decreased expression of all the studied genes below the level observed in salt-stressed plants. However, in some cases, such as POD and tubulin, the expression levels reached values lower than those of the control group.

## Discussion

All living organisms on the planet Earth are currently facing a challenge from climate change, which jeopardizes their survival. It is anticipated that plants' distribution, diversification, and rate of production will change significantly. The planet as a whole would suffer from these changes as a result of increased greenhouse gas emissions, rising temperatures, altering underground water levels, changing precipitation patterns, deteriorating soil texture, and many other severe issues. Specialists must therefore come up with creative approaches that contribute to mitigating the devastating effects of these climate changes to survive these climate changes, in addition to figuring out innovations to the obstacles of food security for the expanding populations. Natural plant extracts have been reported to be an effective treatment for an array of plant stresses, including drought in wheat using moringa extract^23^, fungal invasion in wheat using purslane and chard extracts^15^, salinity stress in soybean using glaucous glasswort extract^9^, and metal stress in flax using moringa extract^24^.

The results of the present study displayed that salt exposition (250 mM NaCl) substantially impaired the growth rate of wheat seedlings as evidenced by the precipitous decrease in shoot height, leaf area, and shoot biomass. Following salt exposure, plants experience alterations in their physiology, biochemical pathways, and molecular pattern. The decreased plant growth triggered by salinity could potentially be due to restricted K^+^ absorption and increased Na^+^ toxicity^25^. The reduced water uptake to shoots, which causes a severe dehydration status in plants^4^, is another probable reason for salt-induced plant growth inhibition. Additionally, salinity impairs cell differentiation and cell cycle, resulting in reduced cell proliferation within plant tissue and a subsequent limitation of expansion^26^. Furthermore, the decreased photosynthesis, transpiration rates, and stomatal conductance following salt exposure disrupts normal physiological processes and nutrient imbalance in plants. The reduced rates of photosynthesis, transpiration, and stomatal conductance by salt exposure also interfere with normal physiological activities and result in nutritional imbalances that hamper plant growth^27^. Additionally, ROS generated under salt stress are cytotoxic and interfere with normal plant metabolism causing oxidative damage, decreased enzyme activity, and disturbed cellular ion homeostasis^28^.

The *Azolla* aqueous extract (AAE) utilized in this investigation stimulated the growth recovery of stressed wheat seedlings. *A. filiculoides* has been widely employed in agriculture for promoting plant tolerance to both abiotic and biotic stress as well as increasing plant growth^29^. According to some investigations, AAE is a rich source of macro- and micronutrients, proteins, jasmonic acid, salicylic acid, and vitamins^30,31^. El-Serafy et al.^29^ evidenced that the presence of such phytochemicals in AAE could boost the levels of endogenous phytohormones and enhance nutrient uptake, resulting in better plant growth and development in both normal and stressful circumstances. The results of the current study showed that AAE when applied foliarly or as a priming treatment, can improve the vegetative growth of salt-stressed wheat seedlings. *A. filiculoides* compost applied to rice plants has been reported to enhance the yield characteristics of rice grown under drought stress as a result of promoting the efficient uptake of nitrogen as well as other nutrients^32^. Furthermore, the considerable amount of nitrogen in AAE, a key component in chlorophyll biosynthesis, was demonstrated to be the primary contributor to the improvement of the growth characteristics of stressed plants following the application of AAE^29^. Also, according to Ibrahim^33^, in alignment with our data, *Azolla* extract applied as a foliar spray or priming treatment lessened the detrimental effects of salt stress on cotton plants. This was because *Azolla* extract improved the biochemical characteristics of the stressed plants, including their pigments, total soluble sugars, total phenolics, carotenoids, protein, amino acids, and antioxidant activities. Consequently, our results demonstrate that the administration of AAE could potentially be a successful tactic for assisting wheat plants to survive in salt-affected fields.

After being exposed to salinity, wheat seedlings exhibited lower chlorophyll concentration as well as reduced photosynthetic activity. The increased chlorophyll breakdown and inhibited chlorophyll synthesis could be the causes of the reduced photosynthetic activity and decreased chlorophyll levels induced by salt effects^34^. The decreased chlorophyll content and photosynthetic activity caused by salt treatment were explained to be a consequence of increased chlorophyllase activity, thylakoid membrane disintegration, chlorophyll-induced oxidative damage, and alterations in chlorophyll-protein complex^4,35^. Moreover, the main cause of the decline in photosynthetic activity was the deactivation of PSII reaction centers brought on by salt stress^36^.

For both control and salt-stressed wheat seedlings, the application of AAE, either as priming or foliar treatments, chlorophyll levels, and photosynthetic activity were increased, principally after priming treatment. These findings lead us to hypothesize that the treatment with AAE increased the availability and uptake of essential nutrients, particularly N and Mg, and facilitated the incorporation of these precursors into the chlorophyll biosynthetic pathway. These results are in agreement with those of^20^ in chard^29^, in quinoa, and^18^ in maize. The high nitrogen content in AAE promotes increased rates of photosynthesis, evaporative transpiration, intercellular CO_2_ concentration, and chlorophyll concentration, subsequently resulting in an elevated net assimilation rate^20,37^.

Concerning carotenoids, NaCl application raised their concentration whereas AAE treatments decreased their level when compared to both normal and stressed controls. AAE treatment also went down the salinity-induced buildup of carotenoids. The increased buildup of leaf carotenoids during salt stress was related to its function as a defense mechanism for the photosynthetic apparatus^38^. The rise in carotenoids could be regarded as a defensive strategy designed to scavenge reactive oxygen, prohibit the oxidation of biomolecules, and prevent the organelles from being deteriorated. Li et al.^39^ reported that the abscisic acid-mediated carotenoids biosynthetic pathway was activated as a protective response against ROS developed under salt stress, enabling plants to develop improved effectiveness in using ROS-eliminating enzymes. The decreased level of carotenoids observed in the present study following AAE application could be ascertained to the high level of carotenoids and other antioxidant compounds in this extract, which provides excellent protection for the growing wheat seedlings under both normal and saline conditions, accordingly, there is no need for its accumulation, and the plant directs its metabolism into the synthesis of other active compounds aiding in the plant growth in a better way.

In addition to ionic toxicity, salinity generates oxidative stress due to the generation of active oxygen species (AOS). This oxidative stress disrupts biological membranes and macromolecules, thereby hampering plant growth and, in extreme situations, might result in death. The results of our study demonstrated that wheat subjected to 250 mM NaCl experienced significant levels of electrolyte leakage (EL), malondialdehyde (MDA), and hydrogen peroxide (H_2_O_2_). The accumulation of photo-reducing energy and discrepancies in the electron transport pathway stimulates the rise in the level of AOS during salt stress. According to^40^, Mehler reaction can be employed to discharge this excessive electrochemical energy, thereby leading to AOS production, including H_2_O_2_, and membrane deterioration, which is reflected in increased EL and MDA levels. Accordingly, disproportionate H_2_O_2_ generation could disrupt the cellular redox potential and advocate membrane leakage as a result of high MDA production.

On the other hand, administration of AAE, either priming or spraying, significantly decreased salt-induced oxidative stress as evidenced by decreased EL values, declined MDA level, as well as decreased H_2_O_2_ accumulation. This result revealed that AAE comprises antioxidant compounds that may function as AOS scavengers against the salt-induced H_2_O_2_, providing safeguarding and stabilization to the cellular membranes of wheat leaves, sustaining their fluidity, and diminishing MDA and EL levels. Many other investigations have demonstrated that *Azolla* extract can promote the growth of stressed plants and reduce the concomitant oxidative stress by lowering MDA, EL, and H_2_O_2_^18,41–43^. The high concentration of secondary active phytochemicals including flavonoids, hormones, alkaloids, polyphenols, terpenoids, amino acids, and fatty acids in AAE contributes to its high antioxidative potential^42^.

To maintain their growth in the face of challenging situations, plants use a variety of adaptation mechanisms. The molecules which accumulate in plants to preserve their osmotic balance by maintaining their hydraulic status are known as osmolytes. Our findings demonstrated that exposing wheat to 250 mM NaCl caused soluble sugars and proline to accumulate while soluble proteins and free amino acid pools significantly decreased. According to^44^, in response to salt stress, proline and soluble sugars function as compatible solutes to decrease the water potential of the plant, creating a water potential gradient that is more appropriate for water uptake and restoring cellular turgidity. Additionally, proline can function as an antioxidant, signaling molecule, and molecular chaperone to defend biomolecules from salt-induced dehydration^45^. According to^46^, soluble sugars do not merely function as the basic components of cells and a source of metabolic energy, but they also operate as signals that modulate several mechanisms associated with plant growth and development. Furthermore, it has been suggested that soluble sugars may act as a chelating agent, causing Na^+^ to be trapped within starch granules contributing to eliminating Na^+^ toxicity in salt-stressed plants^47^. On the other hand, the reported decrease in soluble proteins and free amino acids after salinity stress in our investigation is consistent with the reports of^48,49^. The decrease in soluble proteins and free amino acids driven by salt stress can possibly be speculated to the impairment of nitrate uptake or the irregularity in nitrogen metabolism^50^. The decreased pool of soluble proteins and free amino acids in plants under salt stress can be attributed to defects in the translation process, exhaustion of the carbon skeleton in the formation of antioxidant molecules, denaturation of proteins and amino acids synthesizing enzymes, induction of the proteolytic enzymes, or an imbalance in K^+^, an essential nutrient in the protein synthetic pathway.

In our study, the application of AAE restored the levels of N assimilation in salt-treated wheat seedlings thereby bringing them back to levels that were more or less comparable to the control levels. This may be explained by the phytochemicals in AAE, which provide protection to the metabolic pathways even in stressful situations, enabling stressed plants to sustain their redox and nutritional balances. According to^33^, AAE boosted the growth of cotton plants subjected to salt stress by increasing CO_2_ assimilation, as well as dehydrogenase and nitrogenase activities, which in turn resorted sugar and nitrogenous compound levels.

Salinity stress increases the production of AOS in plant cells and causes membrane lipid peroxidation, protein oxidation, and nucleic acid mutation, all of which suppress plant activities leading to retarded growth. Plants are equipped with defense mechanisms to tackle the issue of oxidative damage. Our work determined that exposing wheat to high salt concentration (250 mM NaCl) increased the activity of the enzymes CAT, POD, APX, PPO, and PAL, while marginally lowering that of SOD. This upregulated antioxidant enzyme activity under saline conditions for the elimination of toxic free radicals has been reported in many investigations^5,51,52^. The elevated activities of the aforementioned enzymes are directly correlated to their role in redox homeostasis to sustain plant endurance under stressful salinity. The increased activity of these enzymes has been reported to improve the growth of stressed plants by safeguarding chloroplasts and other organelle structures, wherein the key biological activities happening^53^. The documented slight decline in SOD activity by salt treatment was observed in six canola genotypes by^54^. This implies that persistent stress reduces SOD activity and makes the detoxification of AOS more challenging.

The results show that when AAE was administered along with salt treatment, the enzymatic antioxidant equilibrium was attained to reach values more or less than the control activities. The high concentration of antioxidant molecules in AAE may be the reason for the reduction in the antioxidant enzyme activity, as the extract offers stressed plants the ability to eliminate AOS, resulting in the antioxidant defense system being downregulated in wheat seedlings. Ibrahim^33^ showed that the advantageous components like vitamins, carotenes, growth promoters, and minerals in AAE could help maximize the antioxidant properties of stressed plants. As a result, AAE can detoxify the AOS on its own without requiring the plant to waste energy protecting itself against oxidative damage.

Salinity causes phenological, anatomical, cellular, metabolic, and molecular alternations, which leads to up- and down-regulation in the expression of some specific genes. Many different genes, such as those involved in energy metabolism, ion transmembranes, photosynthesis, signal transduction, and other metabolic pathways, are associated with salinity stress adaptation. Our results showed that salt stress provoked increased expression of POD, PPO, PAL, PCS (phytochelatin synthase), TLP (thaumatin-like protein), but decreased tubulin (membrane microtubule responsible gene) expression. The upregulated expression of the antioxidant enzymes could be used as a molecular marker reflecting the relationship between the development of salt tolerance and antioxidant activity. H_2_O_2_, a by-product of oxidative stress, has been implicated in the regulation of gene expression in plants exposed to biotic and abiotic stress factors^55^. The greater levels of tolerance to abiotic stressors and the efficient scavenging of AOS were positively linked with the expression of antioxidant enzymes like POD and PPO^56^. PAL is a key enzyme in synthesizing polyphenolic compounds involved in plant response to stresses. Therefore, the increased PAL expression may be related to the production of phenylpropanoids and flavonoids, which act as antioxidant molecules and protect plants from stress by regulating the antioxidant system, photosynthetic apparatus, cellular membrane integrity, and gene expression levels^57^.

A number of studies have demonstrated that PCS expression is upregulated in response to salinity^58–60^. According to those authors, PCS can confer salinity resistance primarily by chelating harmful ions like Na^+^. TLP is an important protein family involved in tissue development and defending plants against biotic and abiotic stresses^61,62^. The overexpression of TLP could be explained by its influence on a group of genes involved in the ABA, ethylene, and auxin signaling pathways to function in protecting against abiotic stress in plants^61^. The tubulin protein family is the main component of microtubules, that constitute the cytoskeleton as a cellular framework within the cytoplasm of cells^63^. Under stressful conditions, increased AOS production led to the downregulation of microtubule-related genes and irreversible damage to the cell structure, leading to a significant impact on cell morphology and functions^64^. Cellular architecture degeneration arises from salt stress-induced quick depolymerization of microtubules and the elimination of cellulose synthase complexes from plasma membranes, which hampers the production of cellulose and the cell wall^65^.

The altered gene expression of the studied genes during the current investigation was comparatively restored following wheat underwent treatment with AAE. This may be ascribed to the extract's related nutritional, antioxidant, and biostimulant profits against the applied salt stress^18^. The downregulation of POD, PPO, PAL, PCS, and TLP gene expression in salt-stressed wheat seedlings by AAE is compelling confirmation that the extract itself is a potent source of antioxidant molecules and therefore that innate immunity is not further integral while dealing with ion toxicity and oxidative stress. As a result, AAE might be a good strategy to reduce the negative effects of salt stress on wheat seedlings. As a result, the decrease in antioxidant enzyme expression brought on by AAE may shed light on the molecular tolerance of wheat seedlings to salt stress.

## Conclusions

Salt stress significantly decreased wheat growth, chlorophyll level, photoassimilation rate, soluble sugars, free amino acids, and tubulin gene expression, while increasing the level of osmoregulators, antioxidant molecules, antioxidative enzymes activity, and stress-responsive genes expression. *Azolla* aqueous extract (AAE) application along with salt treatment enhanced vegetative growth, photosynthetic rate, metabolic efficiency, manipulation of antioxidant enzyme activity, and restoration of stress-related gene expression levels. As a whole, using AAE has the potential to be a useful, affordable, environmentally friendly, and green treatment for mitigating the detrimental consequences of salt stress, particularly when used as a priming treatment. However, to completely grasp the precise mechanism underlying the protective effect of AEE, more research at the proteomic, hormonal, ultrastructural, and molecular levels would be necessary in the future.

## Materials and methods

### Preparation of Azolla extract

The macrophyte *Azolla filiculoides* was harvested from an irrigation canal in Tanta City, Gharbia governorate, Egypt during the spring season of 2022. The harvested plants were carefully washed with tap water and deionized water before being allowed to dry for two days in the shade and then dried at 60 °C in an air-forced oven to a constant weight. The dried plants were ground into a fine powder, sieved using a 2 mm sieve, and then extracted using a mixture of ethanol and water (97.5:2.5 v/v). 200 ml of the extraction solution was added to 5 g of the powder and was subsequently stirred on a rotary shaker for 24 h before being filtered through Whatman No. 1 filter paper. The filtered extract was dried under reduced pressure using a rotary evaporator at 40 °C. The dried powder was collected and then dissolved in distilled water to create the aqueous *Azolla* extract (AAE) stock solution (0.1%). The ready-to-use extract was then stored at − 20 °C in a dark bottle.

### Plant materials and growth conditions

Kernels of bread wheat (*Triticum aestivum* L. cv Sakha 96) were provided by the Agricultural Research Centre, Sakha, Egypt. The kernels were concisely sterilized by drenching for approximately 10 min in 0.1% HgCl_2_. Following a thorough washing with deionized water, the sterilized kernels underwent immersion in two different solutions: deionized water or a solution containing 0.1% AAE for a duration of 21 h. Following the priming time, the kernels were planted in plastic pots (20 × 20 cm) filled with 8 kg of a 2:1 clay-sandy soil mixture. Before applying salt and AAE foliar application treatments, the pots were adequately given tap water until the seedlings had fully emerged (after 6 days). Afterward, the pots were separated into two main groups, with the first group primed with deionized water and the second group primed with AAE as a pretreatment. The water-primed group was split into four sub-treatments: tap water, 250 mM NaCl, AAE spray, and AAE spray + NaCl. However, the AAE-primed group was separated into two sub-treatments: tap water and 250 mM NaCl. Therefore, the experiment included a total of six treatments, each of which was replicated three times in a completely randomized design. The amount of irrigation solution used for each treatment was 70% of the corresponding field capacity according to^1^. AAE was applied onto the upper surface of leaves after the full sunrise using a manual foliar sprayer until full saturation. Throughout the experiment, the spraying process was performed three times at intervals of five days. Plants were harvested after 21 days to evaluate growth and conduct biochemical and molecular measurements.

### Growth evaluation

After plant harvesting, samples were washed with tap water and then deionized water. These samples were measured for shoot height, leaf area, and shoot biomass.

### Leaf pigments and photosynthetic performance (Fv/Fm)

Quantifying the total amount of photosynthetic pigments and monitoring the photosynthetic performance were carried out on the second upper leaf. Fresh foliage samples were extracted using 80% pre-chilled acetone, centrifuged at 7000 rpm (for 5 min), and the absorbance was assessed using a UV/visible spectrophotometer at 663, 644, and 452 for Chla, Chl b and carotenoids, respectively. Following the procedures established by^66^, the fraction of each pigment (Chl a, Chl b, and carotenoids) was calculated and reported as mg g^−1^ FM. The operating efficiency of photosystem II (Fv/Fm) in the dark-adapted leaves was monitored using a portable digital fluorometer (OS-30 P, Hudson, USA) according to^67^.

### Stress-related indicators

Following the technique introduced by^68^, the percentage of leaf electrolyte leakage (EL%) was assessed in wheat fresh leaf discs. After properly washing the leaf discs, they were submerged in deionized water and the electrical conductivity was taken after 15 min. (EC_1_) and after 24 h (EC_2_) and the percentage of EL was calculated.

By using the technique provided by^69^, malondialdehyde (MDA) was identified as a manifestation of membrane oxidative damage triggered by lipid peroxidation. Fresh wheat leaves were extracted in trichloroacetic acid (5%), and the extracts were allowed to react with a solution of thiobarbituric acid (0.67%) in a boiling water bath for 20 min. Following cooling of the mixtures, the absorbance at 530 and 600 nm was determined, and MDA was calculated as nmole g^−1^ FM utilizing the specified molar extinction coefficient (155 mM^−1^ cm^−1^).

Wheat leaves were extracted for hydrogen peroxide (H_2_O_2_) content using a solution of 0.1% trichloroacetic acid (TCA), which was then allowed to react with 1M KI and potassium phosphate buffer (pH 7.0). The mixture's absorbance was determined at 390 nm, and the H_2_O_2_ content (µmol g^−1^ FM) was calculated using the coefficient of 0.28 M cm^−1^.

### Osmoregulatory metabolites

Soluble sugars and proteins in the dry powdered wheat leaves were extracted using borate buffer (pH 8.5). Using the procedure described by^70^, the concentration of soluble sugars was quantified calorimetrically by mixing the borate extracts with conc H_2_SO_4_ and 5% phenol. Glucose used a standard curve to calculate the total soluble sugar concentration. According to^71^, the soluble protein concentration of borate extracts was determined using the Coomassie brilliant blue G250 reagent and a reference protein (Bovine serum albumin).

Free amino acids from wheat leaves were extracted using 80% ethanol. Alcoholic extracts were boiled within a ninhydrin-glycerol-citrate buffer solution for 12 min., after which the absorbance at 570 nm was measured. The quantity of free amino acids was determined by utilizing glycine as a reference amino acid^72^. A 3.0% sulfosalicylic acid solution was used to extract the free proline from the dry-powdered wheat leaves. Sulfosalicylic acid extracts were administered for a 1-h reaction with the acid ninhydrin reagent in boiling water. Toluene was used to take out the performed chromatophore, and its absorbance was detected at 520 nm. The concentration of free proline in wheat leaves was calculated using a standard graph developed using the amino acid proline^73^. All the quantified osmolytes' concentration was expressed as mg g^−1^ DM.

### Enzymatic antioxidants

Fresh leaves of wheat were pulverized in a 100 mM phosphate buffer (pH 7.0) for the extraction of the antioxidant enzymes in this investigation. By implementing a cooling centrifuge set at 4 °C, the extracts were centrifuged at 12,000 rpm for 20 min. The resulting supernatant was subsequently employed for the evaluation of the activities of catalase (CAT), guaiacol peroxidase (POD), ascorbate peroxidase (APX), superoxide dismutase (SOD) and polyphenol oxidase (PPO).

The activities of CAT and POD were investigated by using the assay techniques established by^74^. The CAT assay mixture comprised 0.12 mM H_2_O_2_ within 50 mM phosphate buffer (pH 7.0), and the decline in H_2_O_2_ absorbance was observed at 240 nm throughout 15 min. The POD assay mixture comprised 11.8 mM H_2_O_2_ and 7.2 mM guaiacol within 100 mM phosphate buffer (pH 5.8). The increase in the solution absorbance was recorded at 470 nm.

The method developed by^75^ was used for assessing APX activity. The reaction cocktail consisted of ascorbic acid (0.5 mM), EDTA (0.2 mM), and H_2_O_2_ (0.25 mM) in phosphate buffer (5 mM, pH 7.0). The absorption decrease following H_2_O_2_ dissociation was recorded at 29 nm. SOD reaction was initiated by incorporating the enzyme extract into a reaction vessel which had beforehand comprised 50 mM phosphate buffer (pH 7.8), 10 mM L-methionine, 57 mM nitroblue tetrazolium (NBT), 0.0045% riboflavin, and 0.030% Triton X-100. Under a 30W fluorescent lamp, the reaction vessels were illuminated for 15 min. and the absorbance increment induced by formazan production was monitored at 560 nm^76^.

A 100 mM phosphate buffer solution with 2 mM pyrogallol was used for assaying the activity of PPO. After administering enzyme extract to the assay mixture, the mixture was incubated at 25 °C for 5 min., after which the reaction was stopped by adding 2.5 N H_2_SO_4_. The purpurogallin formation-related increase in absorbance was detected at 420 nm and employed in calculating PPO activity^77^.

Phenylalanine ammonia-lyase (PAL) was extracted from wheat leaves and assayed following the approach adopted by^78^. In brief, wheat leaves were extracted in Tris–HCl buffer (pH 8.8) containing β-mercaptoethanol (15 mM) and the extract underwent a 30-min. cooling centrifugation at 10,000 rpm. For the enzyme assaying, 1 ml extraction buffer, 0.5 ml L-phenylalanine (10 mM), and 0.5 ml enzyme extract were combined and incubated at 37 °C for 1 h then the reaction was terminated by adding 0.5 ml of 4% TCA and the absorbance was read at 290 nm.

Using the corresponding extinction coefficient, the measured enzymes activity was reported as µmol g^−1^ FM min^−1^; the extinction coefficients (mM^−1^ cm^−1^) for the enzymes were 40.0 for CAT, 26.6 for POD, 2.8 for APX, 21.1 for SOD, 26.40 for PPO, and 9.63 for PAL.

### qRT-PCR analysis

Total RNA was extracted using the RNeasy Mini Kit (Qiagen) following the manufacturer's instructions. Complementary DNA (cDNA) was obtained through reverse RNA transcription in a total volume of 20 μl, using a thermocycler (MJ Research, Inc., PTC-100™ Programmable thermal controller, USA). The cDNA reaction conditions included a first enzyme activation cycle at 42 °C for 1 h and a second enzyme inactivation cycle at 80 °C for 15 min.

The SYBR Green PCR Master Mix (Fermentas, USA) was used to perform the qRT-PCR in triplicate. For each reaction, a 25 μl reaction mixture comprising the primer pairs (POD, PPO, PAL, PCS, TLP, and tubulin) (Table 1) was employed, and data were retrieved throughout the extension phase. The reaction was performed using a Rotor-Gene 6000 (QIAGEN, ABI System, USA) using the amplification program prescribed by^79^. The gene β-actin was employed as a reference housekeeping gene for assessing and determining the relative expression of the examined genes^80^.

**Table 1:** qRT-PCR specific primers sequence used in this study.

Gene name	Direction	Sequences 5–3
Peroxidase (POD)	F	GCTTTGTCAGGGGTTGTGAT
	R	TGCATCTCTAGCAACCAACG
Polyphenol oxidase (PPO)	F	CATGCTCTTGATGAGGCGTA
	R	CCATCTATGGAACGGGAAGA
Phenylalanine ammonia-lyase (PAL)	F	AGAACGGTGTCGCTCTTCAG
	R	TGTGGCGGAGTGTGGTAATG
Phytochelatin synthase (PCS)	F	CTTCCAG(A/T)CTCA(G/A)TCGGAGC
	R	ATTGC(G/C)ACTCCT(T/A)GACAGCA
Thaumatin like protein (TLP)	F	CATGTCCTCCCACAGAGTAC
	R	ATATAATCCCATTTCGTGCTTATG
Tubulin	F	AGGATGCTACAGCCGATGAG
	R	GCCGAAGAACTGACGAGAATC
β-actin	F	GTGGGCCGCTCTAGGCACCAA
	R	CTCTTTGATGTCACGCACGATTTC

### Statistical analysis

The results of this investigation were displayed as the average ± the standard deviation of three independent replicates. By using the CoStat software (version 6.311, CoHort), a one-way analysis of variance (ANOVA) was performed to separate the means. The significance of the differences between means was evaluated using the least significant difference (LSD) at the 0.05 level. Except where otherwise stated, *P* < 0.05 was regarded as significant.

### Statement of adherence of the study to IUCN guidelines

The current study complies with relevant guidelines of IUCN Policy Statement on Research Involving Species at Risk of Extinction and Convention on the Trade in Endangered Species of Wild Fauna and Flora.

## Data Availability

All datasets presented in this study are included in the article and can be provided by the corresponding author upon reasonable request.
