# Author Correction: *Azolla filiculoides* extract improved salt tolerance in wheat (*Triticum aestivum* L.) is associated with prompting osmostasis, antioxidant potential and stress-interrelated genes

**DOI:** 10.1038/s41598-024-64033-4

**Published:** 2024-06-06

**Authors:** Asma A. Al-Huqail, Nagwa M. A. Aref, Faheema Khan, Sherien E. Sobhy, Elsayed E. Hafez, Asmaa M. Khalifa, Khalil M. Saad-Allah

**Affiliations:** 1 Chair of Climate Change, Environmental Development, and Vegetation Cover, Department of Botany and Microbiology, College of Science, King Saud University, 11451 Riyadh, Saudi Arabia; 2 Department of Microbiology, Faculty of Agriculture, Ain Shams University, Hadayek Shubra 11241, Cairo, Egypt; 3 Plant Protection and Bimolecular Diagnosis Department, Arid Lands Cultivation Research Institute, City of Scientific Research and Technological Applications, New Borg El‑Arab, 21934 Egypt; 4 Botany and Microbiology Department, Faculty of Science, Al Azhar University (Girls Branch), Cairo, 71524 Egypt; 5 Botany Department, Faculty of Science, Tanta University, Tanta, 31527 Egypt

Correction to: *Scientific Reports* 10.1038/s41598-024-61155-7, published online 15 May 2024

The Acknowledgements section in the original version of this Article was omitted. The Acknowledgements section now reads:

“The authors express their sincere thanks to the City of Scientific Research and Technological Applications (SRTA-City) and the Faculty of Science, Tanta University, Egypt, for providing the necessary research facilities. Also, the authors are grateful to the Deanship of Scientific Research, King Saud University for funding through Vice Deanship of Scientific Research Chairs: Chair of Climate Change, Environmental Development and Vegetation Cover.”

The original Article has been corrected.

